# Retention of health workers in Malawi: perspectives of health workers and district management

**DOI:** 10.1186/1478-4491-7-65

**Published:** 2009-07-28

**Authors:** Ogenna Manafa, Eilish McAuliffe, Fresier Maseko, Cameron Bowie, Malcolm MacLachlan, Charles Normand

**Affiliations:** 1 Centre for Global Health, Trinity College, University of Dublin, Dublin, Ireland; 2 College of Medicine, University of Malawi, Blantyre, Malawi; 3 School of Psychology, Trinity College, University of Dublin, Dublin, Ireland

## Abstract

**Background:**

Shortage of human resources is a major problem facing Malawi, where more than 50% of the population lives in rural areas. Most of the district health services are provided by clinical health officers specially trained to provide services that would normally be provided by fully qualified doctors or specialists. As this cadre and the cadre of enrolled nurses are the mainstay of the Malawian health service at the district level, it is important that they are supported and motivated to deliver a good standard of service to the population. This study explores how these cadres are managed and motivated and the impact this has on their performance.

**Methods:**

A quantitative survey measured health workers' job satisfaction, perceptions of the work environment and sense of justice in the workplace, and was reported elsewhere. It emerged that health workers were particularly dissatisfied with what they perceived as unfair access to continuous education and career advancement opportunities, as well as inadequate supervision. These issues and their contribution to demotivation, from the perspective of both management and health workers, were further explored by means of qualitative techniques.

Focus group discussions were held with health workers, and key-informant interviews were conducted with members of district health management teams and human resource officers in the Ministry of Health. The focus groups used convenience sampling that included all the different cadres of health workers available and willing to participate on the day the research team visited the health facility. The interviews targeted district health management teams in three districts and the human resources personnel in the Ministry of Health, also sampling those who were available and agreed to participate.

**Results:**

The results showed that health workers consider continuous education and career progression strategies to be inadequate. Standard human resource management practices such as performance appraisal and the provision of job descriptions were not present in many cases. Health workers felt that they were inadequately supervised, with no feedback on performance. In contrast to health workers, managers did not perceive these human resources management deficiencies in the system as having an impact on motivation.

**Conclusion:**

A strong human resource management function operating at the district level is likely to improve worker motivation and performance.

## Background

It is widely acknowledged that Africa's health workforce is insufficient and will be a major constraint in attaining the Millennium Development Goals (MDGs) for reducing poverty and disease [1]. The *World health report 2006 *[2] has shown that in general, countries with fewer than 2.3 doctors, nurses and midwives per 1000 people fail to achieve an 80% coverage rate of measles immunization, or the presence of skilled birth attendants during childbirth. Fifty-seven countries fall below this minimum threshold, mainly in sub-Saharan Africa and Asia. This has a major impact on infant and maternal mortality.

A range of factors, including worsening socioeconomic conditions in much of sub-Saharan Africa, increasing mobility and migration of health workers and the absence of strategies to train and retain adequate supplies of appropriate health workers, contributes to the resource drain. The depletion of human resources is particularly acute at the district and community levels, as there are fewer incentives and supports available to attract and retain staff. There is also a lack of understanding of the factors that motivate and attract staff to work at district and community level. In the absence of this information, it is difficult to develop effective human resources strategies.

One of the major challenges facing health systems in sub-Saharan Africa is the international migration of health staff. In addition to international migration there is also considerable in-country migration between the public and private health sectors, between urban and rural areas and between tertiary and primary health care delivery. Increasing flows of health workers into private, urban, tertiary facilities is undermining attempts to provide appropriate public, rural, primary care. For instance, in 2002, Chad's capital, N'Djamena, had 71 doctors per 100 000 people, while in the Charai-Baguirmi region the ratio was only two doctors per 100 000 [3]. In 2002 in Ghana, 55% of pharmacists were in the Greater Accra region, which had 16% of the population, and 2% in the Northern region, with 10% of the population [4].

The main health service provider in Malawi is the Ministry of Health (MOH), which provides approximately 60% of all services. The Christian Health Association of Malawi (CHAM) is responsible for the provision of about 37% of all services. Other providers include both private-for-profit and private, not-for-profit entities, local government, the military and police health services and small clinics offering care for company employees and their families [5].

The shortage of health workers in Malawi is severe even by African standards, with fewer than 4000 doctors, nurses and midwives serving a population of approximately 12 million in 2003. There are 156 physicians working in the Ministry of Health and the Christian Health Association of Malawi. There are 10 districts without an MOH doctor and four districts without any doctor at all [6]. The average number of nurses in health centres is approximately 1.9, an indication that many such centres are run with one nurse or none at all. Fifteen of 26 districts have fewer than 1.5 nurses per facility, and five districts have fewer than one [6].

The human resource (HR) crisis has created a lack of capacity to deliver health services, especially in rural areas where primary health care is severely compromised. Staffing levels are also inadequate for the planned rollout of antiretroviral treatment (ART) and other HIV/AIDS-related services. Essential health package (EHP) scale-up has been critically slowed, with only 10% of the 617 facilities satisfying the HR requirements for delivering EHP in 2003 [5].

In 2005 the Malawi government, with support from donors, initiated the six-year Emergency Human Resources Programme to alleviate the human resources crisis in the health sector. The key components are a salary increase for health professionals; measures to enhance the capacity of training institutions; and, in the short term, additional recruitment of expatriate volunteer doctors and nursing tutors [7]. Of the three components, the salary top-up scheme is designed to improve the working conditions for existing staff, and aims to increase retention of health workers in public service.

In Malawi the majority of health workers are mid-level providers, or cadres of health workers who have shorter training times and who provide services that were originally the preserve of specialists. The documentation and evaluation of these cadres are quite limited, although the few studies exploring their effectiveness have been positive [8,9]. These cadres tend to be paid less than fully qualified doctors and nurses, therefore there are potential economic benefits from their use. If they are not adequately motivated, however, they may migrate out of the health sector or seek employment with NGOs and private sector providers.

In 2007, we undertook a study of three districts in Malawi to map the motivational environment of health workers. A quantitative survey measuring health workers' job satisfaction, perceptions of the work environment and sense of justice in the workplace, reported elsewhere [10,11], found that health workers were particularly dissatisfied with what they perceived as unfair access to continuous education and career advancement opportunities, as well as inadequate supervision.

These issues and their contribution to demotivation, from the perspective of both management and health workers, were explored further by means of qualitative techniques. In addition, we asked both managers and health workers to identify major motivating and demotivating factors and whether they had thought about leaving their current employment. This paper reports the findings from this qualitative part of the study.

**Figure 1:**
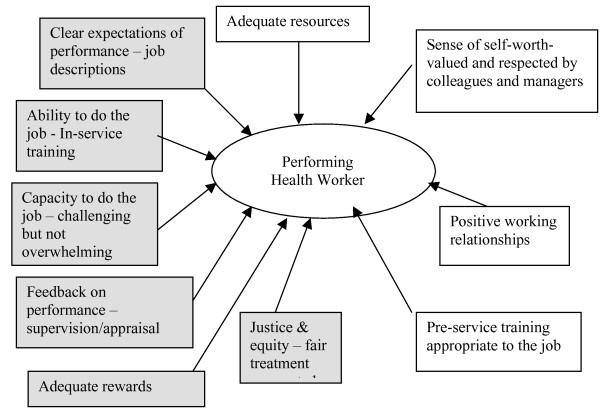
**Factors that contribute to health worker performance**.

This exploratory qualitative study was conducted in the context of a broader human resources study exploring job satisfaction, perceptions of work environment and organizational justice, with the aim of providing evidence to assist in the development of realistic strategies to retain health workers in the districts and improve their performance. Figure 1 identifies the main factors influencing health worker performance that emerged from our research on the perceptions of health workers. This paper focuses particularly on an exploration of the contributory factors on the left hand of the figure, with the other factors in the figure being explored in previous publications on this study.

## Methods

Three districts were purposively sampled from the three geographical regions in Malawi. The main hospital within each district was selected for the focus groups, as this increased the number of staff available to participate. The hospitals selected were: Dowa in the Central region, Thyolo in the South and Karonga in the North. Data for this study were collected in July 2007.

The focus group discussions (FGD) held with health workers were followed by key-informant interviews with district managers and the Ministry of Health. One focus group was held per district, each consisting of seven to 12 participants and lasting between one-and-a-half and two hours. Health workers were selected to capture a diversity of views; participants included: registered nurses, enrolled nurses, clinical officers, medical assistants, assistant environmental health officers, ophthalmology technicians, laboratory technicians, community health nurses, environmental health officers, pharmacy technicians and radiography technicians.

Efforts were made to ensure that the groups were balanced in terms of gender and marital status. Although it is considered good practice for focus groups to be as homogeneous as possible, in this study we were interested in capturing the views of health workers in the districts, and not a specific cadre. We conducted a pilot FGD and from this were confident that mixing the disciplines did not inhibit or skew the discussion, as participants expressed themselves freely in this context. However, health workers indicated during the pilot that district officials should not be included as part of the discussion, as they would not be free to express themselves in their presence.

Nine key-informant interviews were held with members of District Health Management Teams (DHMT) – four with the Human Resource Department of the MOH and two with the Health Service Commission – to explore further issues raised by health workers and to obtain accounts of current human resource policy and practice. Those interviewed within the districts included the District Health Officer, District Nursing Officer and Hospital Administrator. In the MOH the Principal Human Resources Management Development Officer (Training), Principal Human Resources Management Development Officer (Management), Liverpool Associates in Tropical Health Technical Assistant (Training), Liverpool Associates in Tropical Health and Technical Assistant (Management) were interviewed. Two interviews were also held with the Executive Secretary and the Deputy Executive Secretary Health in the Health Service Commission.

The government facilities were chosen because they provide up to 64% of health services in the country and have more challenges with retaining health workers, particularly in rural areas. The focus groups were conducted with a prepared focus group discussion guide and the interviews were semistructured. The analysis of the survey helped inform the contents of the focus group discussion guide and the key informant interviews.

The objectives of the study were explained to participants and confidentiality was assured. Agreement was also obtained to maintain confidentiality within the focus group and not to discuss opinions raised by colleagues outside the focus group setting.

Two research team members conducted the discussion, which explored specific issues surrounding continuous education and in-service training and performance management: supervision/staff appraisal/job description; working conditions; deployment/transfers; and retention factors. Perceptions of what motivates or demotivates these cadres of health workers to work in the public sector were also discussed. Participants were also asked to identify what action the government might take to retain district staff in their posts.

The FGDs and interviews were tape-recorded and transcribed. A thematic analysis employing a framework developed from Figure 1 was used for initial coding. Within each of the thematic areas of the framework, bottom-up coding allowed us to develop a comprehensive picture of issues emerging relating to each particular theme.

## Results

The result of the focus group discussions with health workers and the key-informant interviews with managers are presented together; notable differences of opinion between management/government officials and staff are highlighted where these emerged.

All those who participated in the FGD were permanent staff in full-time employment and had worked in the public sector for at least five years. Minor differences were observed between the various cadres in terms of their opinions on career development and continuing education.

### Clear expectations of performance

All the managers interviewed in the three districts were agreed that current job descriptions did not exist for some cadres of health workers, especially the enrolled nurses and midwives. The job descriptions available to them were outdated. Managers talked about the fact that some staff were not adequately prepared for the roles they were expected to perform.

Most of the health workers indicated that they did not have job descriptions. Those who did have them obtained them from their training colleges. Most of those with job descriptions said they were performing tasks beyond the scope of what was specified in the job description. Those without descriptions said they adapted to the situation and developed an understanding of the expectations from those who had been in post before them.

"We follow what our senior colleagues do and any other (any other task assigned by supervisors), so we are doing more than we are supposed to do".

They found this situation to be frustrating, as they were expected to do more than was specified or than they were trained to do. They believed it was important to be oriented to their jobs before taking up a post.

### Ability to do the job

Health worker training at the level of certificate, diploma or degree is operated by the MOH. The MOH has developed plans for continuous education, but these plans are not always fully funded, due to budget constraints. Recommendation and selection for training is done by the DHMT and ratified by the MOH. All the managers interviewed in the districts and the MOH agreed that continuous education did not necessarily follow government or health needs but was individually driven. This was captured in a statement made by one of the interviewees in the MOH.

"Training needs is on individual basis, it is like you are training and preparing the person for exit from the public sector and the country".

The process of selection for continuous education was considered unfair by health workers. They indicated that opportunities were limited and coordination was lacking. They said that health workers tended to be in service for between eight and 10 years before having access to continuous education. The situation was reported as worse for some cadres, especially the ophthalmic technicians, medical assistants and clinical officers. Environmental Health Officers (including assistants) were the only cadre who indicated that they had obtained training normally within five years.

Health workers also mentioned the lack of rewards for staff who had gained additional qualifications or training as demotivating. An enrolled nurse mentioned that since she completed a diploma more than a year ago, she had not had any promotion or bonus.

The in-service training, which represents training on specific topics to enhance performance, is organized within the districts. Training needs are identified by programme managers and proposals are made to the DHMT for approval. Such training is often organized to fill identified gaps in knowledge in fulfilling patient needs; the process of selection is seen as fair and equitable from the managers' perspective. From the health workers' perspective, in-service training improved their job performance but they mentioned that new skills acquired by staff were sometimes not used. Health workers indicated that they were not paid the amount approved for in-service training in the ministry by the training coordinators. They believed that favouritism seemed also to exist with regard to both continuous education and in-service training. An enrolled nurse said:

"Managers even hide information on training from staff, then they give out the information to the people they like such that sometimes only a set of workers are receiving most of the training."

### Capacity to do the job

Managers acknowledged that the workload within their facilities was high, especially for enrolled nurses and medical assistants in the health centres, and that staffing numbers are not adequate for workloads. They perceived the workload to have negative impacts on staff, as some of them were often agitated and exhausted. In their opinion, this affected their performance and relationship with patients. Thyolo District Health Team observed that because of the high workload some health workers often delegated duties to people not adequately trained for such roles. They had cases where ward assistants were suturing wounds, dispensing drugs and cleaners preparing slides for laboratory technicians. Apart from the problem of medical supplies, most managers interviewed believed the working conditions within their facilities were good. Managers perceived the lack of supplies (equipments and drugs) in the facilities as a major demotivating factor for health workers.

Health workers described their workload as being relatively high and often leading to work stress. An enrolled nurse said:

"Sometimes on night duty I have to cover three or four wards all by myself. This makes me to choose on the ward where I will pay more attention because of the needs of the patients"

They indicated that there was a shortage of staff in almost all the facilities and that the introduction of various new programmes, such as HIV/AIDS treatment, took staff from the existing pool. An enrolled nurse in Thyolo said:

"The HIV clinic increases our workload even though we work with Médecins Sans Frontières in the clinic. We sometimes complain about treating only HIV/AIDS".

They also said that the workload sometimes affected their performance and that when this happened, the council/management perceived it as negligence. Throughout the discussion, health workers complained about the lack of basic supplies to provide adequate care for the patient.

The officials interviewed in the MOH agreed that workload was high but that they had problems with deploying health professionals due to shortages in almost all the cadres. Though deployment was often based on needs, the Ministry did not maintain any standards for deployment. They noted that health workers often did not want to serve in rural districts where basic facilities were lacking.

### Feedback on performance

The DHMT is responsible for supervising staff. Managers interviewed mentioned that they had written standards of performance, but that these did not cover all cadres of health workers. The standards were in the form of a checklist issued from the MoH. They indicated that there were no targets or timelines to allow progress to be measured.

During the FGD health workers expressed dissatisfaction with the supervision they received from management. A nurse said:

"I need to know when I am being supervised and what will be supervised".

In general, health workers felt that management did not give appreciation or recognition for the job they were doing, and this demotivated them. They perceived their professional associations as not being effective in promoting their interests

"Our association is just consuming our money but not protecting our interest. They are there as watchdogs looking out for mistakes".

They also complained of not receiving any feedback from supervisory visits.

When this was discussed with management, the managers agreed that supervision received by staff was often inadequate. The managers felt they were hampered in providing adequate supervision because of their workload. They also evoked their lack of autonomy in creating and following their own supervision standards. One of the DHMT said:

"We do supervise, but most of the standards need to be updated, some items are missing in the checklist".

Another said:

"We are limited in this task because of our workload. We do not have any way of recognizing good performance. We give them a pat on the back and discuss with those not performing'.

The MOH staff interviewed indicated that the Ministry did not have any form of performance appraisal. Two of them were of the opinion that appraising health workers did not make any significant impact on their performance or motivation.

Discussion with health workers suggested limited career progression opportunities. They related this to the absence of a performance appraisal system and a good career structure within the MOH. Health workers were of the opinion that the introduction of an appraisal system would aid managers in making decisions on their career progress. A medical assistant said:

"I have been in this position for the past 13 years without promotion or increment. People that went for their diploma after me now earn more salary than I do. I am so frustrated by this that I have considered resigning even to sell something".

An ophthalmic technician said:

"I have been in this position for the past 11 years; it seems I have been forgotten. The worst of all is that I do not have any opportunity for continuous education".

They expressed concern that promotion opportunities were based on educational qualification only and not on performance. One nurse expressed this as follows:

"Basing promotion on qualification is very wrong. Sometimes you have to wait for 10 years to get further education; that means you remain in the same position for about 10–15 years".

### Adequate rewards

The Ministry of Health in accordance with the Programme of Work increased salaries of health workers (mid-level inclusive) by 52% in 2005. The district health facilities introduced a locum scheme whereby health workers off duty or on holiday could be paid between 600 and 900 Malawi kwacha a day to cover for shortages. The most significant issue that arose for all cadres was salary. They mentioned that their salary was quite poor and did not enable them to meet their individual and family needs. The top-up allowance of 52% did not translate into a 52% increase in take-home pay because of the tax structure in the public service. They indicated that actual increase was within the range of 30% to 35%. A medical assistant said:

"The salary I am paid is too small. I have been a medical assistant for 11 years and I earn the same salary with school leavers".

The locum scheme introduced by the districts was initially seen to be effective, but the impact was diminishing as inflation was rising. Health workers complained that the money had lost value due to inflation and additional needs. The District Health Management Team, especially those in Thyolo, mentioned that they were constantly being approached by staff to increase their locum allowances. From management's point of view, increasing these allowances was not feasible because of funding constraints.

### Justice and equity

Throughout the FGDs there seemed to be several references to the inequities in how staff were treated. A typical example was the inequity in access to training described above. As another example, enrolled nurses expressed their frustration about a change in policy by the Government to offer diplomas instead of certificates to newly graduating enrolled nurses. They indicated that new graduates with diplomas have a better salary and grade on joining the public sector, compared to enrolled nurses with certificates who have served the ministry for a longer period – that is, qualifications are rewarded, but experience is not.

Health workers also expressed their unhappiness with the current Government policy of calling staff for promotion interviews very infrequently and then basing promotion solely on the health worker's performance at the promotion interview, with no account taken of performance on the job.

Sometimes when staff were promoted they were asked to resume the new post in another area, thus forcing people to relocate. They indicated that this relocation was not specified in the advertisements and one was usually told only after being offered the new position. This had resulted in some people's having to live without their families or to forfeit the promotion.

### What do health workers believe motivates them?

Health workers indicated that they were encouraged to take jobs as health professionals within the districts because of the opportunity and ability to assist mankind, coupled with a spirit of patriotism. Health workers were specifically motivated to remain in the districts because of the lower cost of living, the significant impact they made within the communities they served and the fact that they learnt faster on their jobs in the districts compared to their other colleagues in the urban areas. They explained that the limited number of medical officers within the districts meant that they handled difficult and complicated challenges that their colleagues in the urban centres were not allowed to handle.

One major demotivating factor mentioned by all cadres of health workers was monetary. Other demotivating factors mentioned were lack of proper assistance from the Ministry of Health and poor human resource management practices, including lack of supervision and continuous education. In addition, poor housing and the absence of basic amenities such as water and electricity were considered to negatively affect work performance.

### What do managers believe motivates health workers?

Most of the managers believed that health workers were motivated to take up careers in the health sector as a personal choice they had made, the dignity that went with the profession, good career prospects and on humanitarian grounds. Most managers perceived health workers working in their facilities to be moderately motivated. They perceived their motivation to be due to a better salary compared to their colleagues in the teaching profession, better chances for professional development, availability of in-service training, better job security than in the private sector and access to loans and good teamwork. Managers mentioned lack of supply (equipment and drugs) in the facilities; low salary levels for some health workers; lack of promotion or delay in promotion, often of up to five years; high workload; lack of basic amenities such as electricity and water; and problems with accommodationS as major demotivating factors.

### Intention to leave

Of all the managers interviewed, only one indicated that she would have left for the United Kingdom but had to change that decision due to the news she got from those who have migrated outside the country. In her words:

"I was told that houses were expensive and you have to jump from work to work and no rest. I also realized that home is the best, it is better to serve relatives than outsiders and there is reformation in the government, i.e. people are being promoted, improvement in salary, increments and continuous education".

Most of the health workers indicated that they had thought about leaving their job in the public sector in the past year. A clinical officer said:

"Once I finish my internship I will leave the public service to the NGO. My colleagues in the NGO earn MWK 80 000 a month, while I earn MWK 21 000 a month. Though I have better chances to further my education in the public sector, I can still do the same working with the NGO by saving more than half of my salary for two years. My colleague did the same and is back in the university while his mates in the public sector are still waiting for their turn to be trained from the MOH".

A medical assistant indicated his preoccupation with leaving:

"I consider leaving this job on a daily basis, especially since after our former District Health Officer left. I have even thought of going to sell in the market".

From the viewpoint of an enrolled nurse:

"Staying here is not by choice but because of circumstances. I have been applying to NGOS but have not been offered a position by any".

The environmental health officers indicated that they would not want to leave the public sector; one said:

"We have very good chances to further our education within the public sector. Most of my colleagues that graduated before me are already back in school and that is motivating me to stay".

## Discussion

There has been some debate in the literature on motivating and retaining health workers in sub-Saharan Africa [12-14]. These studies have shown that motivation is influenced by both financial and non-financial incentives. Poor salary and working conditions, poor access to training, lack of recognition and lack of adequate performance management systems were the major demotivating factors for health workers. The finding from our FGDs indicated the concern health workers displayed about lack of training, supervision and performance appraisal. Inadequate job descriptions, inadequate supervision and poor regulation and monitoring undoubtedly affect the effectiveness of these cadres of health workers and often result in their carrying out tasks and functions beyond their capabilities – which in turn raises questions about the quality of the care provided. Some studies [15,16] have shown that joint problem-solving between supervisors and health workers is essential for quality improvement and job satisfaction.

Some human resource management activities such as supervision, promotion and training are done as mere rituals with little or no attempt to match needs, while others such as performance appraisal are completely absent. Managers openly admitted to being unable to conduct supervision because of heavy workloads. Dieleman et al. (2006) [13] also found integrated performance management lacking in a study conducted in Mali.

Health workers expressed concern about the lack of career progression, something that is particularly frustrating for clinical officers and medical assistants. The clinical officers undergo four years of training and can progress to medical officer level only by entering the first year of medical school and going through another six years of medical training. Clinical officers feel that they have been trained and forgotten, leaving them without any future prospects.

In Mozambique the introduction of "*tecnico de cirurgia*" was accepted as a temporary solution to a critical problem of scarcity of human resources for health, but no clear attention was paid to the institutional and organizational implications of introducing a cadre playing such an important role. As a result, their career progression was ill-defined [17]. Clinical officers, medical assistants and enrolled nurses who were interviewed said they had few opportunities for refreshing or upgrading their skills. In addition, they found themselves permanently stationed in the rural areas. As the rural areas are where services are needed most, it may be necessary to offer staff opportunities to rotate to peri-urban areas or provide incentives for rural postings or at least introduce transparency in how postings are decided.

From the managers' perspective, their staff were moderately motivated and this was attributed to their employment conditions as health workers relative to the teaching profession. Managers perceived the main demotivating factors to be lack of essential supplies (equipment and drugs) in the facilities, low salary, lack of promotion or delays of up to five years in promotion, high workload and lack of basic amenities, such as basic accommodations serviced with water and electricity. Training, appraisal and supervision did not feature highly in their discussions of demotivation.

The findings of this study indicated that managers and health workers perceived motivation differently. WHO (1993) [18] has also suggested that managers and workers do not necessarily perceive motivation in the same way. It is important that these differences are made explicit, as false assumptions on the part of managers may lead to motivational incentives that do not work for staff.

A particularly worrying finding emerging from this study was that many health workers often considered leaving their jobs. Contrary to the belief that many of these workers will stay within the health system because their qualifications are not internationally recognized (this is the case for enrolled nurses, clinical officers and medical assistants), our findings indicated that NGOs were an attractive option for these health workers because of the higher salaries being offered. Anecdotal evidence suggests that the scarcity of health workers in Malawi prompts NGOs to offer higher salaries than the government in an attempt to attract health workers to the rural clinics, where many of these NGOs operate. This is a serious concern that has received little attention in the published literature and warrants further research to establish the effect of such worker flows on the public health system [19].

## Conclusion

Mid-level health staff described significant demotivating experiences. These need to be addressed in order to maintain these cadres in the public health system: education and training, career paths, scopes of practice and the needs of the workers. The findings highlight the importance of laying down necessary criteria to guide the training and use of health workers. Clear career paths and a continuous education strategy, monitored and evaluated through a functioning, integrated performance appraisal system, are likely to improve staff motivation and retention. This will require a strong human resource management function that operates at the district level.

## Competing interests

The authors declare that they have no competing interests.

## Authors' contributions

OM participated in the literature review, study design and data collection/analysis and drafted this paper. CB participated in the study design and data collection and edited this paper. EM participated in the literature review, study design and data collection/analysis and edited this paper. FM participated in the data collection, data cleaning and preliminary analysis. CN and MM edited the paper. All authors read and approved the final manuscript.
